# Hygiene

**Published:** 1881-09

**Authors:** G. W. A. Lyon


### HYGIENE.

---

Sirs—I have read with considerable interest the article of Dr. Minor on the History of Hygiene, and the more so, in that I hope that a careful persual of these communications will lead some one to an investigation of that too much neglected subject—the study of ancient philosophy. In this matter I quite agree with Dr. Pancost (The Kabbala) that " Little as scientific men of our day know, or are inclined to believe it, the greatest discoveries of the last century and a half can be traced directly to the ancient system of occultism ; few indeed know how much modern science owes to the old mystics." If this be true, the importance of investigation in this line cannot be too highly estimated ; and for thus bringing to our notice the ancient ideas and usages, Dr. Minor deserves the highest praise.

With regard to circumcision : "If we accept the opinions of Dr. Levy (doubtless founded on Gen. xvii., 10), we shall encounter some curious questions. Moses, in spite of Gen. xvii, 14, did not circumcise his offspring, nor did he exercise any careful supervision in this respect over the Israelites during their forty years' wanderings ; and although Yehoeh, or Jehovah, punished discontent with the manna, and the gathering of sticks on the Sabbath (Num. xi.; xv., 32-36). He seems to have been utterly indifferent as to the fulfillment of this expressed command. Consulting Josh. v., we discover that this was left to Joshua, who renewed the right at Gibeah Haaraloth, wherefore the place was called Gilgal—a circle, a heap of stones. For information on this subject, the curious are referred to the cairns, or galgals of various peoples, especially of India.

As to the claim of priority, Herodotus decides in favor of Egypt or of Ethiopia. Indeed, although familiar with Tyre, Babylon, and other places in their vicinity, he never mentions the Jews. Of Babylon he gives a long account ; yet says nothing of the events said to have occurred at that time and place, although Daniel was then scarcely dead. And to anticipate a little, I cannot agree with Dr. Minor that "as ancient a writer as Herodotus maintains that Moses borrowed this idea (prohibition of swine's flesh) from the Egyptians," for I fail to find in the works of Herodotus, or any other ancient writer, the least reference to Moses or a Hebrew nation.

In Book II. 102–4, Herodotus says : "The Colchians, Egyptians and Ethiopians are the only nations of the world who, from the earliest times, have practiced circumcision. For the Phœnicians and the Syrians of Palestine acknowledge that they learned the custom from the Egyptians, and the Syrians about Thermodon and the river Parthenius, with their neighbors, the Macrodes, confess that they very lately learnt the custom from the Colchians ; and these are the only nations that are circumcised, and thus appear to act in the same manner."

After careful investigation, I am fully persuaded that Dr. Minor is correct in his belief that the supporters of the Egyptian origin hold by far the strongest ground ; and so, too, is Bonwick undoubtedly correct in his claim that the rite originated in the phallic element of the sun-myths. In every such myth, among all nations, the sun was born at the beginning of his return northward, at midnight before December 25th. It has made no perceptible advance northward until the 8th day, when his phallus (or vivifying power) was said to be uncovered ; and for this very reason, the year was held to commence with January 1st, instead of its inception on the 8th of the kalends of January. That the rite originated, as others have, from these myths, seems more satisfactory to my mind than that it was instituted for protection and cleanliness, or that God saw that He had made bungling work of His noblest creation.

I have said that other ceremonies and ideas arose from the sun-myths, and Dr. Minor's remarks on "pork" give an instance. The hog was an emblem of winter, or the evil principle, Typhon or Set, who was opposed to Osiris, the Sun and Horus, the Sun, the Savior ; and the eighth chapter of Matthew has its counterpart in Egyptian mythology. The boar was represented in the heavens by the Great Bear, or the Ass of Typhon, which is in the ascendent when the sun is in Libra, preparatory to its descent into Sheol, or the grave of winter. In Grecian mythology, it is the Erymantian Boar, slain by Hercules, also a sun-god ; and as if to make the identity complete, they tell us that Callisto was changed into a bear, and placed by Jupiter in the sky as the constellation Ursa Erymanthis, Ursa Major, or Great Bear.

Respectfully,

G. W. A. LYON, M.D., in *Lancet and Clinic.*

